# Treatment of Herpes Labialis: Comparison of Two OTC Drugs and Untreated Controls

**DOI:** 10.1111/j.1708-8240.2011.00417.x

**Published:** 2011-04-27

**Authors:** James P McCarthy, William D Browning, Craig Teerlink, George Veit

**Affiliations:** * Vice President of Research and Regulatory Affairs, Quadex Pharmaceuticals, LLC7070 Union Park Center, #380, Midvale, UT 84047, USA; † Professor and Indiana Dental Association Endowed Chair in Restorative, Indiana University School of DentistryDS S-317, 1121 W Michigan St., Indianapolis, IN 46202, USA; ‡ Research Instructor, Division of Genetic Epidemiology, Department of Internal Medicine, University of Utah391 Chipeta Way, Ste D, Salt Lake City, UT 84108, USA; § Chairman—Department of Dental Hygiene, Utah Valley University987 South Geneva Road, Provo, UT 84058, USA

## Abstract

**Statement of the Problem:**

Rapid resolution of active herpes labialis lesions is of great benefit to the patient not only in terms of controlling pain and disfigurement, but in disruption of needed dental treatment.

**Purpose of the Study:**

Using three groups, this retrospective study investigated the time required to complete healing and the loss of discomfort.

**Methods and Materials:**

Based on 180 completed surveys, responses were divided into three groups: One group used Abreva (GlaxoSmithKline, Parsippany, NJ, USA). The second used Viroxyn (Quadex Pharmaceuticals, West Jordan, UT, USA). The third group, the Control group, consisted of untreated lesions. All three groups were asked about past experiences with lesions treated using Abreva and/or Viroxyn, and lesions which were left untreated. In addition, 58 participants who had used Viroxyn only responded. Participants were provided standardized responses from which to choose.

**Results:**

For both the time to healing and time to loss of discomfort, participants in both the Abreva and Viroxyn groups experienced significant improvements. Relative to the Abreva, Viroxyn provided significant improvement in both outcomes (all *t*-tests; all *p* < 0.001). Relative to the Control group, Viroxyn and Abreva offered an 8.0 and 4.0 day reduction in time to healing, respectively. Loss of discomfort occurred within 3.0 days and 1 hour for Abreva and Viroxyn, respectively.

**Conclusion:**

Relative to the untreated controls, both Abreva and Viroxyn offered a significant reduction in both the time to healing and time to loss of discomfort. Furthermore, Viroxyn offered a significant reduction relative to Abreva.

**CLINICAL SIGNIFICANCE:**

Providing care to patients with active lesions greatly increases the risk of spread of infection from one site to another and to members of the dental team. Precautions include the use of special personal protective equipment and limiting treatment during active outbreaks to urgent care only. Accordingly, any treatment that shortens healing time for active lesions would contribute significantly to the health and well-being of the patient and the dental team.

(J Esthet Restor Dent 24:103–111, 2012)

## INTRODUCTION

### Herpes Labialis (Cold Sore/Fever Blister)

Recurrent herpes labialis, typically caused by herpes simplex virus type-1 (HSV-1), is a condition affecting nearly 40% of the US population.^1^ Although in most people the disease is self-limiting, it is critical to note that the “common cold sore” is not always a trivial disease. Recent studies have established that over 70% of new diagnoses of genital herpes in young people are caused by HSV-1.^2^ HSV-1 can result in serious consequences in the immune incompetent.

Whereas some people experience 12 to 13 lesions per year,^3^ 4 lesions per year is typical. Untreated lesions heal in about 10 days.^4^ In a comprehensive review of the clinical literature, Esmann's findings were similar.^5^ The typical progression is: (1) a prodromal period, (2) a papule, (3) a vesicle, and (4) an ulcer. Healing begins with formation and subsequent loss of a scab.^4^ Physical discomfort is greatest during the progression from papule through ulcer, with reports of pain, itching and burning peaking during open ulcer stage.^4^,^6^

### Treatment Options and Standard of Care

Current treatments include Rx drugs or over-the-counter (OTC) skin protectants and/or analgesic products. See Table 1 for a list of health care provider treatment options as found in the dental literature.^7^,^8^

**Table 1:** Antiviral drugs for herpes simplex virus

Systemic use drugs	

Generic name (brand name)	*Suggested dosage × duration (days)

Acyclovir (Zovirax)	400 mg tid × 7

Famciclovir (Famvir)	125 mg bid × 5

Valacyclovir (Valtrex)	500 mg bid × 5

Topical use drugs	

Generic name (brand name)	*Suggested dosage × duration

Penciclovir (Denavir) cream 1%	Every 2 hours

Acyclovir (Zovirax) cream 5%	Every 2 hours

Docosonol cream, 10% (Abreva†)	Every 3 hours

IPA Tincture of BZK, 0.13% & 5% Benzocaine†	

(Viroxyn Solution)	Single application

* Variations in dosage and time are adjusted for clinical severity.

*Essentials of oral medicine* (2001) Silverman S, Eversole L, & Truelove E, BC Decker, Hamilton, ON. Tables 13-2, p. 122. Reprinted with permission.

† These items were added to the table to improve consistency by providing a generic and brand name descriptions for all drugs listed.

Prescription drugs are only effective if begun very early, and reduce healing time by 1 to 2 days.^5^ They have several drawbacks: (1) time to healing is reduced by only 10%, (2) unwanted side effects, (3) they are expensive, and (4) the delay in scheduling an office visit is likely to render use of the drug ineffective.

Only two, Abreva (GlaxoSmithKline, Parsippany, NJ, USA) and Viroxyn (Quadex Pharmaceuticals, West Jordan, UT, USA), are OTC drugs. Abreva is a 22-carbon long-chain fatty alcohol. Product claims are that the fatty alcohol acts to inhibit viral migration by providing a hindrance. It is applied three times per day, with the number of days of application being adjusted to the severity of the lesion. Viroxyn is believed to disrupt the lipid viral envelope using a classical surfactant action. It is applied in one single application.

The purpose of this study is to investigate the time required to complete healing and the relief of pain in three groups—the Abreva group, the Viroxyn group, and the Control group. The hypothesis that there is a significant difference between groups will be tested against the null hypothesis of no difference at a significance level of 5%.

### Rationale for Study Design

The “gold standard” for a drug study typically includes multiple study sites, randomization, double blinding,and placebo control. As a result of multimillion-dollar advertising campaigns, it is probably impossible to achieve blinding with OTC products; a trip to the drugstore or a few moments on the internet are all that is needed to identify the drug.

The design of this study conforms to the newly issued Food and Drug Administration (FDA) Guidance.^9^,^10^ These guidelines contain several key provisions: First, the study participant must directly report the outcome metrics on a suitable instrument without input or filtering from any health care professional. Second, there must be a reasonable expectation that the participant has a good memory of the outcome. Third, the measurements under study are clinically relevant to the disease and can be evaluated by a layman. The present protocol was submitted to the FDA prior to the start of the study and was approved without comment following a 30-day review process. The results of the study will be submitted to the FDA, separate from any publication. Still, retrospective studies have the potential for several types of design bias, and factors identified as potential sources of bias are identified and examined in the Discussion section.

## METHODS AND MATERIALS

Using the three groups of participants identified, this study investigated two primary outcomes. Again, two groups used the OTC drugs Abreva and Viroxyn. The third, the Control group, consisted of the responses of participants regarding untreated cold sore lesions of which the participant had a clear memory. Specifically, the primary outcome objectives studied were:

1. Time to persistent resolution of discomfort (pain, itching, and burning).
2. Time to healing (loss of hard scab and return to intact skin).

Everyone who purchased the Viroxyn online was sent a survey and asked to participate in the study. To encourage participation, respondents were provided with a complimentary three-pack of Viroxyn.

As it was assumed that some participants would have experience with the Abreva, the survey also included questions about these experiences. Similarly, it was assumed that participants would be able to recall lesions that went untreated, and questions were included about them as well. All surveys were returned directly to the investigators and data entries were made by one person and verified by a second person. Surveys that included data for Viroxyn, but not for Abreva, were segregated and analyzed separately.

### Survey

First participants were asked to provide their initials for identification purposes, basic demographic data, and an estimate of how many cold sores they experience per year. Next participants were asked about their experiences with untreated lesions. Then, they were asked to indicate the time to healing (loss of hard scab and return to intact skin) and the number of days until the pain was rated as mild. Answers were provided in whole numbers. Where participants recorded a range, the lowest number reported was used.

Participants were next asked the same questions about their experience with the Viroxyn. Finally, they were asked if they had also used Abreva. If so, they answered the same questions about their experience with it. To standardize responses for these two drugs, the time intervals were presented in a multiple choice format. For the time-to-healing data, the participants were offered choices from 1 day to “10 days or more.” For this outcome, responses were in whole numbers.

For the time to loss of pain, participants were offered the following responses:

1. 2 minutes (0.00139 days);
2. 10 minutes (0.0069 days);
3. 1 hour (0.042 days);
4. 12 hours or less (0.5 days);
5. 1 day;
6. 2 to 4 days;
7. 4 to 6 days; and
8. More than 6 days.

### Statistical Analysis Plan

All participants who returned a survey with at least one data point showing outcome data for either drug were included in the analysis. Simple summary statistics (mean and standard deviation) were provided for demographical information. Differences in age between male and female participants were analyzed using a *t*-test.

Again, this study was conducted using the design preferred by the FDA. In conformance with the preferences of the FDA, for both of the primary outcomes, the Control, Abreva, and Viroxyn groups were compared using *t*-tests. The data were not normally distributed and more typically would be analyzed using an analysis of variance (ANOVA) on ranks procedure. To assure the choice of testing did not change the results, additional testing was performed. The type of test did not affect the results (ANOVA on ranks and signed rank tests; all results *p* < 0.05).

Data from those participants who had used Viroxyn but not Abreva are presented as an additional observation. Using *t*-tests, these data were analyzed separately against reports of untreated lesions within the Viroxyn only group.

With regard to simple descriptive statistics, most scholarly papers on this topic describe time-to-healing outcomes in terms of the median value. The median is used because it tends to negate the effects of outlying data and because the data are often not normally distributed. In this study, the data were not normally distributed due to study design ceilings on reporting time-to-healing data and time to loss of discomfort. To satisfy norms of statistical testing and the preferences of the FDA, the study outcome data are presented both as median value and as mean value with the standard deviation in parentheses.

## RESULTS

### Participant Reported Outcome Results

Three hundred and sixty-nine surveys were sent out, and 32 were returned as undeliverable. One survey was returned with no data recorded. Of the 336 usable surveys, 238 were returned for a response rate of 71%. Table 2 shows that the distribution of responses strongly favors those who had experience with both products. Demographic data are also presented in Table 2. The demographics of the group with experience using both products favored women over men, and Caucasians over other races, but the differences in ages of the groups were not statistically significant.

**Table 2:** Distribution of responses

Viroxyn and Abreva users	*N* = 180	75.6%
	
	Median	Mean (SD)

Number of cold sores per year	4.0	4.7 (3.4)

Demographic information		

Male	*N* = 69	38.3%

Age in years mean (SD)	40.8	(12.6)

Females	*N* = 111	61.7%

Age in years mean (SD)	41.1	(11.9)

Comparison of age: male versus female		*p* = 0.86*

Ethnicity		

Caucasian	*N* = 170	94.4%

Other or missing	*N* = 10	5.6%

Viroxyn only users	*N* = 58	24.4%
	
	Median	Mean (SD)

Number of cold sores per year	4.0	4.2 (2.7)

Demographic information		

Male	*N* = 26	44.8%

Age in years: mean (SD)	44.6	(15.5)

Females	*N* = 32	55.2%

Age in years: mean (SD)	42.3	(13.3)

Age: male versus female		*p* = 0.6*

Ethnicity		

Caucasian	*N* = 53	91.4%

Other	*N* = 5	8.6%

* *t*-test.

### Participants Experienced Using Both Products—Primary Outcome Results

The number of cold sores per year (Table 2) is consistent with the literature values.^1^,^3^–^5^

Median and mean scores for time to healing are reported in Table 3. Use of both Abreva and Viroxyn resulted in a significant decrease in time to healing. Furthermore, participants who used Viroxyn healed in significantly fewer days than did those who used the Abreva (all results of *t*-tests; all *p* < 0.001).

**Table 3:** Days until healed

	Median	Mean (SD)
Viroxyn and Abreva users		

Control	11.0	11.5 (3.8)*

Abreva	7.0	7.6 (2.2)†

Viroxyn	3.0	4.0 (2.0)‡

*N* = 180		

Viroxyn only users		

Control (untreated cold sore)	10.0	11.1 (3.3)*

Viroxyn	3.0	4.0 (2.5)†

*N* = 58		

Definition: loss of hard scab and return to intact skin.

* Control versus Abreva (*t*-test; *p* < 0.001).

† Control versus Viroxyn (*t*-test; *p* < 0.001).

‡ Viroxyn versus Abreva (*t*-test; *p* < 0.001).

Median and mean scores for time to loss of discomfort are reported in Table 4. Use of both Abreva and Viroxyn resulted in a significant decrease in time to loss of discomfort. Furthermore, participants who used Viroxyn had significantly fewer days of discomfort than did those who used Abreva (all results from *t*-test; *p* < 0.001).

**Table 4:** Days until loss of discomfort

	Median	Mean (SD)
Viroxyn and Abreva users		

Control	6.0	6.7 (3.5)*

Abreva	3.0	2.8 (2.1)†

Viroxyn	0.042	0.6 (1.0)‡

	(1 hour)	(14.4 hours)

*N* = 180		

Viroxyn only users		

Control	5.0	6.3 (3.7)

Viroxyn	0.052	0.007 (1.2)†

	(1.3 hours)	(10 minutes)

*N* = 58		

Definition: consistent categorization of pain as being mild-none.

* Control versus Abreva (*t*-test; *p* < 0.001).

† Control versus Viroxyn (*t*-test; *p* < 0.001).

‡ Viroxyn versus Abreva (*t*-test; *p* < 0.001).

### Participant Who Have Used Viroxyn but Never Used Abreva

The number of cold sores per year (Table 2) is also consistent with the literature values,^1^,^3^–^5^ and the demographics data are similar as well (Table 2).

The data demonstrate that the healing time for the Control or untreated lesions in the group that had only used Viroxyn were very similar to the experiences of the untreated lesions reported by participants who had used both drugs. It was also consistent with the literature.^5^ Relative to the Control group (Table 3), participants who used Viroxyn experienced a significant reduction in time to healing (*t*-test; *p* < 0.001). For the time to loss of discomfort outcome, the pattern of the data was similarly consistent with untreated controls. Relative to the Control group (Table 4), participants who used only Viroxyn experienced a significant reduction in time to loss of discomfort (*t*-test; *p* < 0.001).

## DISCUSSION

### Unmanageable Study Biases of a Prospective Study

As noted, due to the universal availability of both study drugs in the OTC marketplace, blinding study operators and/or participants would be impractical. Any institutional review board would require that all brand names be revealed. Thus, there would be no way to assure that participants were not affected by previous advertisements or the opinions of acquaintances. Given the unique differences in appearance, odor, and method of application of the two drugs, no clever scheme to disguise the drugs would work. Thus, a retrospective study using the guidelines established by the FDA was more credible.

### Weaknesses of Retrospective Studies

Participants' responses were based on memories of their experience with untreated cold sores (Control) and with the use of Viroxyn and/or Abreva. This is a weakness of retrospective studies. However, it is important to note that in this study, memory problems would affect both groups equally.

Another weakness of retrospective studies is that participants are not chosen at random. These participants, for example, were identified because they had purchased Viroxyn online. Accordingly, one might have concerns about how representative these participants were of average cold sore sufferers. Similarly, since Abreva is more well known and 75% of those surveyed had used both drugs, one might wonder if these participants represented a subset of people with lesions that were more difficult to treat, i.e., had used Abreva with less than satisfactory results and chose to try Viroxyn the next time. The demographic data and the data on the Control group do not support this conclusion. Rather, they indicate that these participants are consistent with those in the literature. The demographics between those who had used both and those who had only used Viroxyn are consistent (Table 2). Similarly, Control group data are consistent with those who reported that they had used both drugs and those who had not used Abreva (Tables 3 and 4). Furthermore, to the extent that they might represent more difficult cases, the challenge they presented would be equivalent for both drugs. It should be noted that the participants are predominately Caucasian in all groups. The authors are not aware of any papers that claim a difference in response to herpes based on ethnicity.

### Study Design-Induced Bias Favors Abreva

For the time to healing and time to loss of discomfort, data were censored or cut off at an artificial point. For healing responses of “10 days or more,” a value of 10 was assigned. Similarly, discomfort data reported as “more than 6 days” were assigned the value of 6 days. In terms of healing, a large number of participants reported this response: Abreva (*N* = 63 or 35.0% of respondents) and Viroxyn (*N* = 2 or 1.1% of respondents). Since values greater than 10 were not recorded, the data for Abreva tended to underestimate the time to healing. This represents a real bias that worked in favor of Abreva. To a smaller extent, this was also true for the discomfort data. The Abreva group (*N* = 24 or 13.3% of respondents) had more data points censored than the Viroxyn group (*N* = 1 or 0.6% of respondents).

### Bias Related to Price, Availability, and Stature

The participants paid $16 to $20 for Abreva at the drugstore and immediately had their purchase in hand. In contrast, participants paid over $40 for Viroxyn online and waited for delivery. Although both drugs are lawfully marketed OTC drugs, only Abreva may describe itself as the “Number 1 Cold Sore Medication Recommended by Pharmacists.” All of these issues tend to create a more favorable impression of Abreva. In short, Abreva is much more well known and accepted. For Viroxyn to be shown as equivalent, despite these biases, would be noteworthy. In the present study, Viroxyn was shown to be more effective.

All respondents were offered a complimentary sample of Viroxyn at the time they were asked to complete the survey, i.e., before their use and level of satisfaction with either Viroxyn or Abreva were known to the authors. Potentially, consumers who were more satisfied with their use of Abreva rather than Viroxyn may have been more likely not to participate. This would result in a bias toward Viroxyn. As cited above, the data do not indicate that there was any important difference between groups. Rather, the demographic data for the untreated control lesions for both Abreva and Viroxyn users are equivalent and in line with reports in the literature.

## CONCLUSIONS

Within the limitations of the study, it can be concluded that the use of Viroxyn was associated with a significantly shorter mean healing time compared to Abreva, 4.0 days versus 7.6 days. Also, both medications resulted in a significantly shorter mean healing time relative to untreated Control lesions. Conclusions regarding days until loss of discomfort parallel that of the healing time with Viroxyn resulting in a significant decrease (0.6 days) compared to Abreva (2.8 days). Again, both groups performed significantly better than the Control group (6.7 days).
